# Supplementary material for: Therapeutic Education for Safer Rheumatologic Care: A Scoping Review to Map Evidence on Infection Prevention
Source: Nurs Rep. 2025 Dec 4;15(12):431. doi: 10.3390/nursrep15120431 (PMC12735640; doi:10.3390/nursrep15120431)
Supplement: Supplementary file 1 [file nursrep-15-00431-s001.zip › Table S1.pdf]

Supplementary Materials

Table S1. Example search string

PubMed search string
(Rheumatic diseases[Title/Abstract] OR (Rheumatic[Title/Abstract] AND musculoskeletal disease[Title/Abstract]) OR rheumatology OR systemic sclerosis[Title/Abstract] OR Juvenile idiopathic arthritis [Title/Abstract] OR Rheumatoid Arthritis [Title/Abstract] OR arthritis, juvenile [Title/Abstract] OR juvenile idiopathic arthritis [Title/Abstract] OR reactive arthritis[Title/Abstract] OR osteoarthritis [Title/Abstract] OR spondyloarthropathies [Title/Abstract] OR spondylitis ankylosing [Title/Abstract] OR polymyalgia rheumatica [Title/Abstract] OR mixed connective tissue disease [Title/Abstract] OR lupus erythematosus, cutaneous [Title/Abstract] OR systemic [Title/Abstract] OR sjogren's syndrome [Title/Abstract] OR vasculitis [Title/Abstract] OR myositis [Title/Abstract] OR fi[Title/Abstract] OR autoimmune diseases [Title/Abstract] OR Title/Abstract] OR autoimmune diseases [Title/Abstract] OR Connective tissue disease [Title/Abst act] OR Rheumatoid vasculitis [Title/Abstract] OR Psoriatic arthritis [Title/Abstract]) AND (Disease modifying antirheumatic drug [Title/Abstract] OR antirheumatic agents[MeSH Terms] OR Synthetic DMARDs [Title/Abstract] OR methotrexate [Title/Abstract] OR azathioprine [Title/Abstract] OR leflunomide [Title/Abstract] OR ciclosporin [Title/Abstract] OR cyclophosphamide [Title/Abstract] OR sulfasalazine [Title/Abstract] OR hydroxychloriquine [Title/Abstract] OR infliximab [Title/Abstract] OR adalimumb [Title/Abstract] OR Certolizumab [Title/Abstract] OR Golimumab [Title/Abstract] OR Canakinumab [Title/Abstract] OR Rituximab [Title/Abstract] OR Tocilizumab [Title/Abstract] OR Sarilumab [Title/Abstract] OR Secukinumab [Title/Abstract] OR Ustekinumab [Title/Abstract] OR Belimumab [Title/Abstract] OR ixekizumab [Title/Abstract] OR Etanercept [Title/Abstract] OR Abatacept [Title/Abstract] OR Anakinra [Title/Abstract] OR TOFACITINIB [Title/Abstract] OR BARICITINIB [Title/Abstract] OR UPADACITINIB [Title/Abstract] OR FILGOTINIB [Title/Abstract] OR APREMILAST [Title/Abstract] OR Biosimilar biologic DMARDs [Title/Abstract] OR Biologic DMARDs [Title/Abstract] OR Ts- BDMARDs [Title/Abstract]) AND (Health promotion[Title/Abstract] OR Health knowledge [Title/Abstract] OR Health education[Title/Abstract] OR Health prevention [Title/Abstract] OR patient education[Title/Abstract] OR promoting safety skills[Title/Abstract] OR safety competencies [Title/Abstract] OR educating patient [Title/Abstract] OR education session[Title/Abstract] OR education program [Title/Abstract]) AND (Prevention of infections [Title/Abstract] OR risk of infection [Title/Abstract] OR (risk factors [Title/Abstract] AND infections [Title/Abstract])).
